# Physical activity and DNA methylation–based markers of ageing in 6208 middle-aged and older Australians: cross-sectional and longitudinal analyses

**DOI:** 10.1007/s11357-024-01408-5

**Published:** 2024-11-07

**Authors:** Haoxin Tina Zheng, Danmeng Lily Li, Makayla W. C. Lou, Allison M. Hodge, Melissa C. Southey, Graham G. Giles, Roger L. Milne, Brigid M. Lynch, Pierre-Antoine Dugué

**Affiliations:** 1 Cancer Epidemiology Division, Cancer Council Victoria, Melbourne, VIC Australia; 2 Precision Medicine, School of Clinical Sciences at Monash Health, Monash University, Clayton, VIC Australia; 3 Centre for Epidemiology and Biostatistics, Melbourne School of Population and Global Health, The University of Melbourne, Parkville, VIC Australia; 4 Department of Clinical Pathology, The University of Melbourne, Parkville, VIC Australia

**Keywords:** Biological ageing, Epigenetic ageing, Lifestyle, Physical activity, Longitudinal data

## Abstract

Epigenetic age quantifies biological age using DNA methylation information and is a potential pathway by which physical activity benefits general health. We aimed to assess the cross-sectional and longitudinal associations between physical activity and epigenetic age in middle-aged and older Australians. Blood DNA methylation data for 6208 participants (40% female) in the Melbourne Collaborative Cohort Study (MCCS) were available at baseline (1990–1994, mean age, 59 years) and, of those, for 1009 at follow-up (2003–2007, mean age, 69 years). Physical activity measurements (weighted scores at baseline and follow-up and total MET hours per week at follow-up) were calculated from self-reported questionnaire data. Five blood methylation–based markers of ageing (*PCGrimAge*, *PCPhenoAge*, *bAge*, *DNAmFitAge*, and *DunedinPACE*) and four fitness-related markers (*DNAmGaitspeed*, *DNAmGripmax*, *DNAmVO2max*, and *DNAmFEV1*) were calculated and adjusted for age. Linear regression was used to examine the cross-sectional and longitudinal associations between physical activity and epigenetic age. Effect modification by age, sex, and BMI was assessed. At baseline, a standard deviation (SD) increment in physical activity was associated with 0.03-SD (*DNAmFitAge*, 95%CI = 0.01, 0.06, *P* = 0.02) to 0.07-SD (*bAge*, 95%CI = 0.04, 0.09, *P* = 2 × 10^−8^) lower epigenetic age. These associations were attenuated after adjustment for other lifestyle variables. Only weak evidence was found for the longitudinal association (*N* = 1009) of changes in physical activity and epigenetic age (e.g. *DNAmFitAge*: adjusted *β* =  − 0.04, 95%CI =  − 0.08, 0.01). The associations were not modified by age, sex, or BMI. In middle-aged and older Australians, higher levels of self-reported physical activity were associated with slightly lower epigenetic age.

**Supplementary Information:**

The online version contains supplementary material available at 10.1007/s11357-024-01408-5.

## Background

Physical inactivity is a major risk factor of many chronic diseases, shorter life span [1, 2], and unhealthy ageing trajectories [3]. Ageing, the progressive deterioration of tissues over time, leads to impaired physical functioning and increased risks of morbidity and mortality [4, 5]. Markers of biological age may provide a useful quantification of risk of disease [4]. People of the same chronological age may have different biological age due to differences in genetics and adverse exposures (e.g. environmental, lifestyle) accumulated during their lifetime [6]. In recent years, ‘epigenetic clocks’, based on DNA methylation (DNAm), have emerged as powerful markers of biological age [7]. Higher values of epigenetic age have been reported to be associated with increased risk of many age-related diseases [8–10].

The few studies that have examined the cross-sectional association between physical activity and epigenetic age have produced mixed results, with some studies reporting negative associations [11–13], and others weak or no association [14–17]. Kresovich et al. found that 1 h additional recreational physical activity per week was associated with 0.4-year lower *GrimAge* and no reduction for *HannumAge*, *HorvathAge*, or *PhenoAge* [11]. Fox et al. reported negative, non-linear associations of physical activity with *GrimAge* and *PhenoAge* [12]. A 2-year four-armed randomised controlled trial found a reduced number of stochastic epigenetic mutations, but no changes in *GrimAge*, among 56 women assigned to a regular exercise intervention compared with 58 age-matched controls [18].

Additional to these inconsistencies across studies, all used a cross-sectional design and mainly considered the first- and second-generation clocks (*HorvathAge*, *HannumAge*, *PhenoAge*, *GrimAge*). Several additional measures have been developed in recent years, mainly (i) principal component (PC)–based clocks, which provide more reliable versions of *PhenoAge* and *GrimAge* [19], and (ii) incorporating additional information about inflammation (*bAge*, [20]) and physical fitness (*DNAmFitAge*, [21]), and (iii) measuring the rate of ageing (*DunedinPACE*, [22]) instead of biological age at a given point in time. Additionally, we hypothesised that the effect of physical activity on epigenetic age may be different by sex, age, and BMI, based on evidence that males and females vary in both their levels of physical activity [23] and biological ageing profiles [24], that older adults tend to be less physically active than younger adults [25], and the bi-directional link between obesity and physical activity [26].

This study aimed to improve our understanding of the potential pathways through which physical activity positively impacts health, and the value of novel methylation-based markers in this context, by assessing (i) whether physical activity is associated with epigenetic age; (ii) whether this association varies by sex, age, and BMI, in a large sample of middle-aged to older Australians.

## Methods

### Study sample

We used data from the Melbourne Collaborative Cohort Study (MCCS), a cohort of 41,513 middle-aged and older Australians (99% aged between 40 and 69 years, 59% females) of white European origin recruited in 1990–1994 (baseline) [27]. A face-to-face follow-up was carried out in 2003–2007 [27]. At both waves, demographic, lifestyle and health data, physical measurements, and blood samples were collected [27].

The MCCS was approved by the Human Research Ethics Committee of the Cancer Council Victoria, Melbourne, VIC, Australia, and informed consent was provided by all participants according to the Declaration of Helsinki.

### Physical activity variables

Physical activity data was collected using self-administered questionnaires about frequency of different types of activity (walking, vigorous, and non-vigorous activities) at baseline and using the International Physical Activity Questionnaire short-form (IPAQ-short) at follow-up [27, 28]. Physical activity scores were calculated by accounting for walking, non-vigorous activity, and vigorous activity, with vigorous activity being assigned twice the weight of the other categories [29]. At baseline, the score (maximum range, 0–16) was based on the frequency of each type of activity over the past 6 months (for each activity type: 1: none, 1.5: 1–2 times per week, 4: ≥ 3 times per week), while at follow-up, the score (maximum range, 0–168) was based on the duration (hours/week) spent on each type of activity in the past 3 months. At follow-up, the total metabolic equivalent of task (MET) hours per week, which integrates the weekly frequency, duration, and intensity of physical activities, was also calculated by multiplying the time spent on each type of activity by its corresponding MET value and summing these [27, 30].

The distribution of the physical activity measures was strongly right-skewed and these were therefore log-transformed as ln (physical activity score + 1). For a better comparison of results across the two waves and calculation of changes in physical activity between time points, we standardised the physical activity measures to *z*-scores. The change in physical activity between the two waves was calculated as the standardised physical activity score at follow-up minus the standardised baseline score.

### DNA methylation

A total of 7688 samples (including duplicates) were available from participants in eight nested cancer case–control studies, where incident cancer cases were identified through annual linkage to the Victorian Cancer Registry and were matched to controls on age, sex, and country of birth (and smoking history for lung cancer study) using incidence density sampling, Supplementary Fig. 1. For the longitudinal analyses, we used a subset of 1100 controls from six out of eight of these case–control studies who were randomly selected from all controls and attended both baseline and follow-up visits [27]. DNAm data were then processed using the Illumina HumanMethylation450 BeadChip array [27]. The same normalisation and quality control procedures were applied to all methylation data [27]; the detailed methods have been described previously [31, 32].

### Epigenetic ageing variables

We considered primarily five epigenetic measures of ageing as outcomes: *PCPhenoAge*, *PCGrimAge*, *bAge*, *DNAmFitAge*, and *DunedinPACE. PhenoAge* was developed based on the DNAm surrogate marker of phenotypic age that includes ten clinical markers [33]. *GrimAge* was developed with DNAm markers of smoking pack-years and seven plasma proteins to predict all-cause mortality [34]. These were trained on PCs derived from DNAm data to improve their reliability, resulting in *PCPhenoAge* and *PCGrimAge* [19]. Both *bAge* and *DNAmFitAge* were developed based on *GrimAge*, where *bAge* incorporated 6/8 DNAm-based surrogate markers from *GrimAge* and 28 protein *EpiScores* (e.g. C-reactive protein and many cytokines) [20], and *DNAmFitAge* combined *GrimAge* with three DNAm markers of fitness-related variables: gait speed (*DNAmGaitspeed*), maximum hand grip strength (*DNAmGripmax*), and maximum volume of oxygen intake (*DNAmVO2*_*max*_) [21]. *DunedinPACE* was calculated based on DNAm-based markers of changes in 19 biomarkers over 20 years to estimate the rate of ageing [22]. We additionally considered the individual four methylation-based predictors of fitness variables: *DNAmGaitspeed*, *DNAmGripmax*, *DNAmVO2*_*max*_ and DNAm-based forced expiratory volume 1 (*DNAmFEV1*) [21].

All epigenetic ageing measures and DNAm-based predictors of fitness-related variables were calculated using the *methscore* function in R [35]; *bAge* and *DNAmFitAge* were calculated based on *PCGrimAge*. The residuals from regressions of the epigenetic markers on chronological age were calculated to obtain age-independent measures of biological age; these were further standardised to *z*-scores and used in all analyses.

### Covariates

The covariates were collected via questionnaires and physical measurements at baseline and follow-up, including age, sex (male, female), country of birth (Australia/New Zealand, Northern Europe, Southern Europe), smoking status (never, former, current smoker), smoking pack-years (log-transformed), socioeconomic status (socioeconomic index for areas [SEIFA] [36], decile), alcohol consumption (g/day), body mass index (BMI, kg/m^2^), and diet quality (Alternative Healthy Eating Index 2010 [AHEI-2010], calculated from food frequency questionnaires).

### Data preparation

After excluding duplicates and participants with missing data in physical activity score or covariates, baseline data were available for 6208 participants. At follow-up, after excluding participants who did not have data on physical activity measures, some participants had missing data across confounders (0.1–7.0%, Supplementary Table 1). These were imputed using the *missForest* method which performs well with mixed data types and a small proportion of missing data [37, 38].

### Statistical analysis

Pearson’s correlation coefficients were calculated between epigenetic age measures, physical activity scores at both waves, and MET score at follow-up. Descriptive statistics of the crude epigenetic markers are provided in Supplementary Table 2.

Linear regression models were used to assess the cross-sectional and longitudinal associations between physical activity and epigenetic age. For each model, potential non-linearity was assessed using restricted cubic splines (3 knots at the 10th, 50th, and 90th percentiles), by comparing models with linear or spline terms using likelihood ratio tests (LRTs). In the case of non-linearity, spline graphics were drawn. Three models were fitted: Model 1 adjusted for age, sex, and country of birth; Model 2 additionally adjusted for SEIFA score, smoking status, smoking pack-years, alcohol consumption, and AHEI-2010; Model 3 additionally adjusted for BMI. Cross-sectional analyses were performed separately at baseline (*N* = 6208) and follow-up (*N* = 1009) using covariates at each corresponding wave. At follow-up, physical activity was assessed using both physical activity score and MET hours/week.

For longitudinal analyses of the association between changes in physical activity scores and follow-up epigenetic age, two models with confounders measured at baseline were considered: Model 1: age at follow-up, sex, country of birth, baseline epigenetic age, and baseline physical activity; Model 2: additionally adjusted for SEIFA score, smoking status and pack-years, alcohol consumption, AHEI-2010, and BMI.

In a previous study, we found a U-shaped association between weight change and epigenetic age [39]. In addition, a non-linear association between alcohol consumption and various health outcomes has been widely reported [40]. Therefore, BMI and alcohol consumption were modelled using restricted cubic spline terms (3 knots at 10th, 50th, and 90th percentiles).

#### Secondary analyses

The same cross-sectional and longitudinal analyses were repeated for the four DNAm-based predictors of fitness-related markers. We also repeated the cross-sectional analyses for each type of physical activity (walking, non-vigorous, and vigorous activity).

#### Sensitivity analyses

We tested for effect modification by age, sex, and BMI by comparing Model 3 (fully adjusted, cross-sectional associations at baseline) with and without interaction terms between physical activity and sex, age (continuous) and BMI (continuous), respectively, using LRTs. Subgroup analyses for (i) males, females; (ii) younger adults (age < median age, 60 years), older adults (age ≥ 60 years); and (iii) normal/underweight (BMI < 25 kg/m^2^), overweight (25 kg/m^2^ ≤ BMI < 30 kg/m^2^), obese (BMI ≥ 30 kg/m^2^) were performed to illustrate the results.

Analyses were conducted with Stata 16.1 and R 4.3.2.

## Results

### Sample characteristics

The mean age of the 6208 participants (40% females) at baseline was 58.8 years, with a median physical activity score of 4.0 (interquartile range [IQR], 1.5–5.5), Table 1. At follow-up, 1009 participants were included, with 31% females and a mean age of 68.6 years. The median physical activity score was 4.7 (IQR, 2.0–9.5) and the median MET score was 17.3 (6.9–34.7) hours/week. For baseline variables in the baseline sample (*n* = 6208) and in the subset of participants who also had data at follow-up (*N* = 1009), most of the lifestyle-related factors including physical activity were similar across the two samples (e.g. BMI, baseline sample: mean = 27.2; subset: mean = 26.6). Some differences were noticed regarding epigenetic profiles (e.g. *PCPhenoAge*, baseline sample: mean =  − 0.08; subset: mean = -0.84).

**Table 1:** Characteristics of the study participants from the Melbourne Collaborative Cohort Study

Variables	Cross-sectional analysis (*N* = 6208)	Longitudinal analysis (*N* = 1009)	
	Baseline	Baseline	Follow-up
Age, mean (SD)	58.8 (7.7)	57.3 (7.9)	68.6 (8.0)
Sex, *N* (%)			
Female	2504 (40.3%)	313 (31.0%)	
Male	3704 (59.7%)	696 (69.0%)	
Country of birth, *N* (%)			
Australia/New Zealand	4229 (68.1%)	787 (78.0%)	
Northern Europe	403 (6.5%)	91 (9.0%)	
Southern Europe	1576 (25.4%)	131 (13.0%)	
SEIFA score, median (IQR)	6 (3, 8)	7 (4, 9)	7 (4, 9)
Smoking status, *N* (%)			
Current smoker	862 (13.9%)	102 (10.1%)	54 (5.4%)
Former smoker	2367 (38.1%)	392 (38.9%)	440 (43.6%)
Never smoked	2979 (48.0%)	515 (51.0%)	515 (51.0%)
Smoking pack-years^a^, mean (SD)	3.0 (3.1)	2.8 (3.0)	
Alcohol consumption, median (IQR)	4.3 (0, 17.1)	7.5 (0, 19.1)	9.1 (0, 23.1)
BMI (kg/m^2^), mean (SD)	27.2 (4.1)	26.6 (3.7)	27.0 (4.1)
AHEI-2010, mean (SD)	65.4 (14.2)	63.8 (11.3)	52.5 (10.3)
Walking^a^, median (IQR)	1.5 (0, 4)	1.5 (0, 4)	3.0 (1.3, 6.0)
Vigorous physical activity^b^, median (IQR)	0 (0, 0)	0 (0, 0)	0 (0, 0.5)
Non-vigorous physical activity^b^, median (IQR)	0 (0, 1.5)	0 (0, 1.5)	0 (0, 0.5)
Physical activity score^b^, median (IQR)	4 (1.5, 5.5)	4 (1.5, 7)	4.7 (2.0, 9.5)
MET score, median (IQR)			17.3 (6.9, 34.7)
Change in physical activity score^c^, mean (SD)			− 0.08 (1.2)
*PCPhenoAge*, mean (SD)	− 0.08 (6.57)	− 0.84 (5.12)	0.18 (5.90)
*PCGrimAge*, mean (SD)	− 0.15 (3.87)	− 0.16 (3.19)	− 0.03 (3.38)
*bAge*, mean (SD)	− 0.02 (0.46)	− 0.05 (0.39)	− 0.002 (0.44)
*DNAmFitAge*, mean (SD)	− 0.09 (4.74)	− 1.07 (4.35)	0.04 (4.43)
*DunedinPACE*, mean (SD)	− 0.003 (0.13)	− 0.02 (0.12)	0.00 (0.12)
*DNAmGaitspeed*, mean (SD)	0.00 (0.10)	0.01 (0.10)	− 0.001 (0.09)
*DNAmGripmax*, mean (SD)	− 0.23 (7.90)	1.69 (7.51)	0.16 (7.51)
*DNAmVO2max*, mean (SD)	− 0.05 (2.30)	0.75 (1.97)	0.02 (1.86)
*DNAmFEV1*, mean (SD)	− 0.01 (0.59)	0.14 (0.56)	0.01 (0.45)

^*^IQR, interquartile range; *SD*, standard deviation; *SEIFA*, socioeconomic index for areas; *BMI*, body mass index; *AHEI-2010*, Alternative Healthy Eating Index 2010; *MET*, Metabolic Equivalent of Task; *VO2amx*, maximum volume of oxygen consumption; *FEV1*, forced expiratory volume in 1 s

^**^All epigenetic ageing measures were age-adjusted

^a^The smoking pack-years variable was log-transformed and not available at follow-up

^b^At baseline, each type of physical activity was assessed as ‘number of times’ per week: 0 = no activity, 1.5 = 1–2 times per week; 4 = 3 or more times per week and combined into an overall score (range, 0–16), with double weight for vigorous physical activity. At follow-up, physical activity was assessed as a duration (hours/week) spent on each types of physical activity (double weight for vigorous activity, range, 0–168), and METs

^c^Change in physical activity score was calculated as the difference between *z*-scores of ln (physical activity score + 1) at baseline and follow-up

At both waves, the physical activity scores were more strongly correlated with walking (e.g. follow-up: *r* = 0.82, Supplementary Figs. 2 and 3) than with vigorous or non-vigorous physical activity (e.g. vigorous activity: *r* = 0.52).

### Cross-sectional analyses

There was no evidence that any of the associations of physical activity with epigenetic age deviated from a linear association (LRT: P_linearity_ > 0.05 for all models). At baseline, in Model 1, 1-SD higher physical activity score was associated with 0.03-SD (*DNAmFitAge*, *β* =  − 0.03, 95%CI =  − 0.04, − 0.01, *P* = 0.02) to 0.07-SD (*bAge*, *β* =  − 0.07, 95%CI =  − 0.09, − 0.04, *P* = 2 × 10^−8^) lower epigenetic age, Table 2. In Model 2, these effect estimates were null for *DNAmFitAge* (*β* =  − 0.001, 95%CI =  − 0.03, 0.02), and weakly negative for *PCGrimAge*, *bAge*, and *DunedinPACE* (e.g. *bAge*: *β* =  − 0.03, 95%CI =  − 0.05, − 0.01). Adding BMI slightly attenuated the associations (e.g. *bAge*: *β* =  − 0.02, 95%CI =  − 0.04, 0.00). At follow-up, only *PCGrimAge*, *bAge*, and *DunedinPACE* showed similar associations with physical activity / MET score in Model 1 (e.g. MET score, *bAge*: *β* =  − 0.07, 95%CI =  − 0.12, − 0.01) and all associations were closer to null in fully adjusted models, with effect estimates similar to the baseline analyses based on a larger sample of participants. *PCPhenoAge* and the four markers of fitness-related variables were not associated with physical activity scores at either wave (e.g. *PCPhenoAge*, Model 1 at baseline: *β* =  − 0.01, 95%CI =  − 0.04, 0.01).

**Table 2:** Cross-sectional associations between physical activity and epigenetic markers of ageing and fitness-related variables at baseline (*N* = 6208) and follow-up (*N* = 1009)

PA measures	DNAm-based ageing markers	Model 1^a^			Model 2^b^			Model 3^c^		
		*β*	95%CI	*P*	*β*	95%CI	*P*	*β*	95%CI	*P*
Baseline PA score (*N* = 6208)	*PCPhenoAge*	− 0.01	− 0.04, 0.01	0.30	− 0.02	− 0.04, 0.01	0.22	− 0.01	− 0.04, 0.01	0.38
	*PCGrimAge*	− 0.06	− 0.09, − 0.04	**9 × 10**^**−7**^	− 0.03	− 0.05, − 0.00	**0.02**	− 0.02	− 0.05, − 0.00	**0.03**
	*bAge*	− 0.07	− 0.09, − 0.04	**2 × 10**^**−8**^	− 0.03	− 0.05, − 0.01	**0.01**	− 0.02	− 0.04, − 0.00	**0.03**
	*DNAmFitAge*	− 0.03	− 0.06, − 0.01	**0.02**	− 0.001	− 0.03, 0.02	0.91	0.001	− 0.02, 0.03	0.93
	*DunedinPACE*	− 0.06	− 0.09, − 0.04	**7 × 10**^**−7**^	− 0.03	− 0.06, − 0.01	**0.01**	− 0.02	− 0.04, 0.01	0.15
	*DNAmGaitspeed*	0.001	− 0.02, 0.03	0.95	− 0.01	− 0.04, 0.01	0.27	− 0.02	− 0.04, 0.01	0.21
	*DNAmGripmax*	− 0.001	− 0.01, 0.01	0.71	− 0.003	− 0.01, 0.00	0.49	− 0.003	− 0.01, 0.00	0.41
	*DNAmVO2max*	0.004	− 0.02, 0.02	0.73	− 0.01	− 0.03, 0.01	0.56	− 0.01	− 0.03, 0.01	0.50
	*DNAmFEV1*	0.002	− 0.00, 0.01	0.50	0.002	− 0.00, 0.01	0.50	0.002	− 0.00, 0.01	0.51
Follow-up PA score (*N* = 1009)	*PCPhenoAge*	− 0.03	− 0.09, 0.03	0.30	− 0.02	− 0.08, 0.04	0.56	− 0.01	− 0.07, 0.05	0.75
	*PCGrimAge*	− 0.06	− 0.12, − 0.01	**0.03**	− 0.03	− 0.07, 0.02	0.29	− 0.02	− 0.07, 0.03	0.37
	*bAge*	− 0.06	− 0.12, − 0.00	**0.05**	− 0.02	− 0.07, 0.03	0.47	− 0.01	− 0.06, 0.04	0.72
	*DNAmFitAge*	− 0.03	− 0.09, 0.03	0.39	− 0.003	− 0.06, 0.06	0.92	− 0.005	− 0.05, 0.06	0.87
	*DunedinPACE*	− 0.06	− 0.12, − 0.00	**0.04**	− 0.03	− 0.09, 0.03	0.30	− 0.01	− 0.06, 0.05	0.79
	*DNAmGaitspeed*	− 0.01	− 0.07, 0.06	0.85	− 0.02	− 0.08, 0.04	0.58	− 0.02	− 0.09, 0.04	0.45
	*DNAmGripmax*	− 0.001	− 0.02, 0.02	0.91	− 0.002	− 0.02, 0.02	0.82	− 0.003	− 0.02, 0.01	0.73
	*DNAmVO2max*	− 0.003	− 0.05, 0.04	0.89	− 0.01	− 0.05, 0.04	0.78	− 0.01	− 0.06, 0.04	0.62
	*DNAmFEV1*	0.01	− 0.00, 0.03	0.09	0.01	− 0.00, 0.03	0.16	0.01	− 0.01, 0.03	0.20
Follow-up MET score (*N* = 1009)	*PCPhenoAge*	− 0.03	− 0.10, 0.03	0.28	− 0.02	− 0.08, 0.04	0.54	− 0.01	− 0.07, 0.05	0.75
	*PCGrimAge*	− 0.07	− 0.12, − 0.01	**0.02**	− 0.03	− 0.08, 0.02	0.22	− 0.03	− 0.07, 0.02	0.30
	*bAge*	− 0.07	− 0.12, − 0.01	**0.02**	− 0.02	− 0.07, 0.02	0.34	− 0.01	− 0.06, 0.04	0.58
	*DNAmFitAge*	− 0.03	− 0.09, 0.03	0.37	− 0.003	− 0.06, 0.06	0.92	0.01	− 0.05, 0.06	0.86
	*DunedinPACE*	− 0.08	− 0.14, − 0.02	**0.01**	− 0.04	− 0.10, 0.02	0.15	− 0.02	− 0.07, 0.04	0.56
	*DNAmGaitspeed*	− 0.01	− 0.07, 0.06	0.85	− 0.02	− 0.08, 0.04	0.57	− 0.02	− 0.09, 0.04	0.44
	*DNAmGripmax*	− 0.001	− 0.02, 0.02	0.88	− 0.002	− 0.02, 0.02	0.80	− 0.004	− 0.02, 0.01	0.70
	*DNAmVO2max*	− 0.01	− 0.05, 0.04	0.77	− 0.01	− 0.06, 0.04	0.68	− 0.02	− 0.06, 0.03	0.51
	*DNAmFEV1*	0.02	− 0.00, 0.03	0.05	0.01	− 0.00, 0.03	0.10	0.01	− 0.00, 0.03	0.13

^a^Model 1 adjusted for age, sex, and country of birth

^b^Model 2 additionally adjusted for SEIFA score, smoking status, smoking pack-years, AHEI-2010, and alcohol consumption (spline term)

^c^Model 3 additionally adjusted for BMI at follow-up (spline term)

^*^All epigenetic ageing measures were age-adjusted and were standardised to a mean of 0 and standard deviation of 1

^**^All physical activity scores were log-transformed and were standardised to a mean of 0 and standard deviation of 1

### Longitudinal analyses

There was weak evidence of an association between changes in physical activity and epigenetic ageing markers in any of the models, Table 3. In Model 2, the effect estimates per SD were − 0.03 (95%CI =  − 0.08, 0.01) for *bAge* and − 0.05 (95%CI =  − 0.11, 0.01) for *DunedinPACE*. A weak association was found for *DNAmFEV1* (Model 2: *β* = 0.02, 95%CI = 0.00, 0.04).

**Table 3:** Longitudinal associations between changes in physical activity over a decade and epigenetic markers of ageing and fitness-related variables at follow-up (*N* = 1009)

DNAm-based ageing markers	Model 1^a^			Model 2^b^		
	*β*	95%CI	*P*	*β*	95%CI	*P*
*PCPhenoAge*	− 0.03	− 0.09, 0.02	0.26	− 0.02	− 0.08, 0.03	0.42
*PCGrimAge*	− 0.04	− 0.08, 0.01	0.09	− 0.03	− 0.08, 0.01	0.13
*bAge*	− 0.03	− 0.08, 0.01	0.14	− 0.03	− 0.07, 0.02	0.24
*DNAmFitAge*	− 0.04	− 0.10, 0.02	0.21	− 0.03	− 0.09, 0.03	0.34
*DunedinPACE*	− 0.05	− 0.11, 0.01	0.10	− 0.03	− 0.09, 0.03	0.28
*DNAmGaitspeed*	0.01	− 0.06, 0.07	0.88	− 0.01	− 0.07, 0.06	0.86
*DNAmGripmax*	0.01	− 0.01, 0.03	0.47	0.01	− 0.01, 0.03	0.56
*DNAmVO2max*	0.004	− 0.05, 0.06	0.88	0.001	− 0.05, 0.05	0.96
*DNAmFEV1*	0.02	0.00, 0.04	**0.02**	0.02	0.00, 0.04	**0.03**

^a^Model 1 adjusted for age, sex, country of birth, baseline epigenetic age/DNAm fitness markers, and baseline physical activity

^b^Model 2 additionally adjusted for SEIFA score, smoking status, smoking pack-years, AHEI-2010, alcohol consumption (spline term), and BMI (spline term)

^*^All epigenetic ageing measures were age-adjusted, and were standardised to a mean of 0 and standard deviation of 1

^**^Change in physical activity during follow-up was calculated as the difference between standardised physical activity scores at baseline and follow-up, and was standardised to a mean of 0 and standard deviation of 1

### Types of physical activity and epigenetic age

At baseline, in Model 1, fairly strong negative associations with epigenetic age were found for vigorous activity (e.g. *bAge*: *β* =  − 0.07, *P* = 9 × 10^−8^, Supplementary Table 3), and weaker for walking (e.g. *bAge*: *β* =  − 0.03, *P* = 0.02), and non-vigorous activity where an association was only observed for *bAge* (*β* =  − 0.03, *P* = 0.02). After adjusting for lifestyle-related factors, vigorous activity showed some association with *bAge* (*β* =  − 0.02, *P* = 0.03) and *DunedinPACE* (*β* = -0.03, *P* = 0.01), with minimal attenuation in BMI-adjusted models (*bAge*: *β* =  − 0.02; *DunedinPACE*: *β* = 0.02). At follow-up, there was a negative association for walking with epigenetic age (e.g. *bAge*: *β* =  − 0.06, *P* = 0.04) in Model 1 and all associations were null in more adjusted models.

### Subgroup analyses

**Fig. 1:**
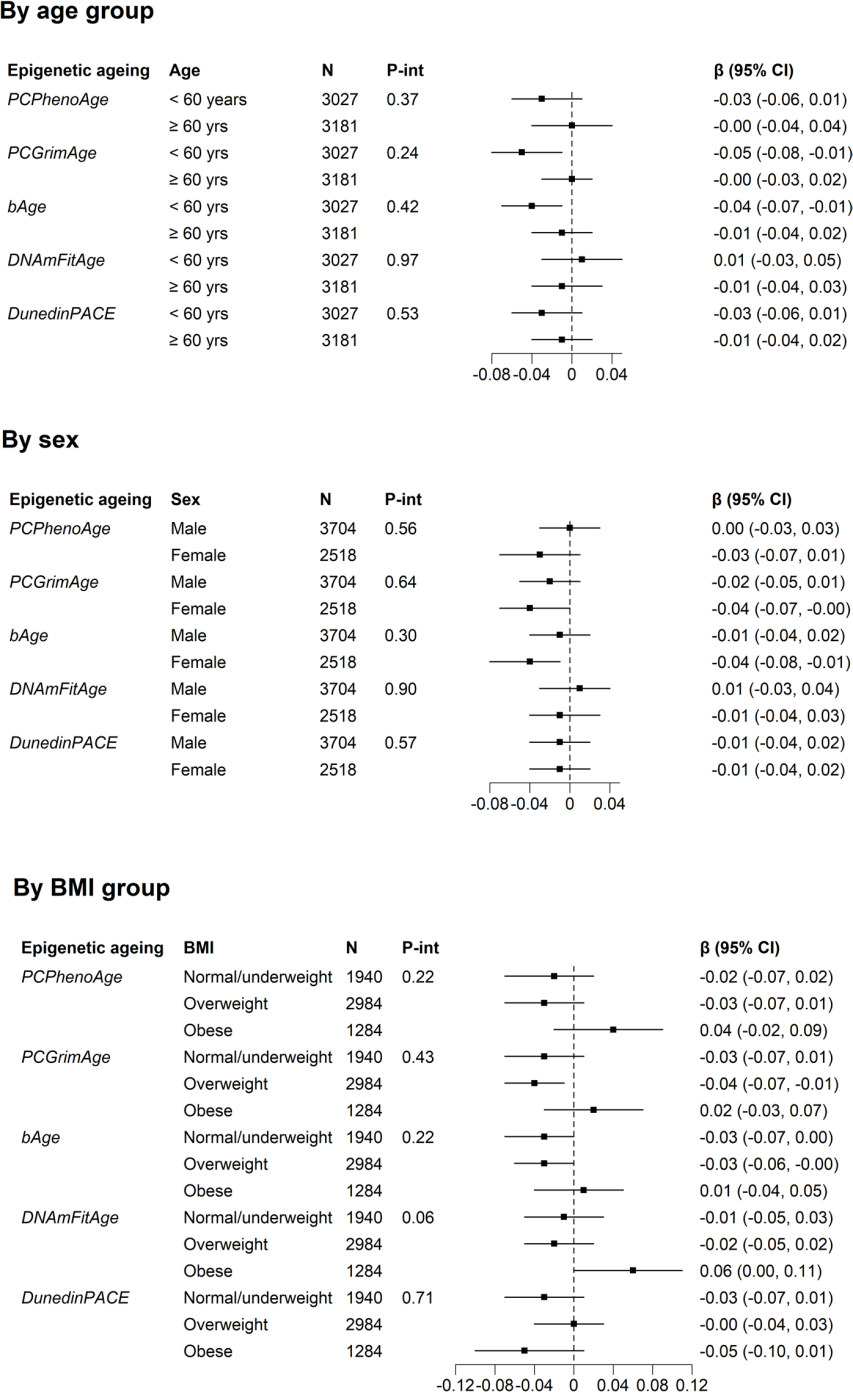
Subgroup analyses of the cross-sectional (baseline) and longitudinal associations between physical activity and epigenetic age by age, sex, and BMI*.* *Results from Model 3, adjusting for age, sex, country of birth, SEIFA score, smoking status, smoking pack-years, AHEI-2010, alcohol consumption (spline term), and BMI (spline term, not included in the subgroup analyses by BMI). **BMI was divided into two groups: normal/underweight: BMI < 25 kg/m^2^, overweight: 25 kg/m^2^ ≤ BMI < 30 kg/m^2^, and obese: BMI ≥ 30 kg/m.^2^. ***All epigenetic ageing measures were age-adjusted and standardised to a mean of 0 and standard deviation of 1. ****All physical activity scores were log-transformed and standardised to a mean of 0 and standard deviation of 1

There was no evidence of effect modification by age, sex, or BMI (P_interaction_ ≥ 0.05 for all epigenetic markers), Fig. 1. There was some suggestion that inverse associations were marginally stronger for younger adults, females, or normal/underweight participants than their respective counterparts, particularly for *PCGrimAge* and *bAge*.

## Discussion

This study examined the cross-sectional and longitudinal associations between physical activity and epigenetic age. Cross-sectionally, higher physical activity levels were associated with slightly lower levels of epigenetic markers of ageing, although most associations appeared to be due in part to other characteristics of physically active/inactive participants. Little evidence was found for associations of self-reported physical activity with epigenetic markers of fitness-related variables. The results for longitudinal analysis over approximately a decade were overall consistent but based on a smaller number of participants.

In previous studies, strong associations with epigenetic age have been found for various lifestyle-related factors including smoking, diet, and obesity [16, 39], and the associations between physical activity and epigenetic age were substantially attenuated after adjusting for these factors [11, 14]. Lifestyle-related factors are related to physical activity in various ways: e.g. smokers are more likely to be physically inactive than non-smokers [41]; active people are more likely to have healthy dietary habits and vice versa [42]; increased physical activity may reduce weight [43], and obesity may be a barrier for engaging in physical activity [44]. Elliot et al. [43] reported that a combination of improving physical activity and diet was associated with greater health improvements than improving exercise habits or diet alone. While we found no interaction with age, sex, or BMI in our study, physical activity might be more effective in slowing ageing when combined with other lifestyle modifications, such as weight management, dietary improvements, and smoking cessation.

In our study, the association with physical activity appeared somewhat stronger for *bAge* (and *PCGrimAge*). *bAge* incorporates DNAm markers for various proteins related to inflammation, particularly C-reactive protein [20] which is known to be associated with physical activity [45], and is a key risk factor for disease, including cancer [46]. Although *DNAmFitAge* and the four related markers of fitness-related variables were developed to reflect physiological fitness [21], the associations were close to null in our study. In a previous publication [21], the associations of physical activity with *DNAmFitAge* and sub-markers varied across cohorts and were no greater than with *GrimAge* and *PhenoAge*. We nevertheless acknowledge that measures of physical fitness would be a valuable addition to study the effects of physical activity on DNAm-based surrogate markers such as *DNAmFitAge*, *DNAmFEV1*, and *DNAmVO2max* and biological ageing more generally, as done in previous studies [47, 48], but these were not available in our study.

Several studies have assessed the association between physical activity and epigenetic age. Using 2818 participants from the MCCS, Dugué et al. [16] found no evidence of associations of physical activity with *HorvathAge* or *HannumAge*. Broadly consistent results were also reported in 2435 participants from the Framingham Heart Study Offspring cohort, with a negative association for accelerometer-measured physical activity was observed for *GrimAge* [14]. Fiorito et al. [15] pooled data from 5111 participants in 18 cohorts and found that low physical activity level was associated with 0.22-year older *HorvathAge* compared to high activity level. Using 2758 participants from the Sisters Study, the associations between physical activity and epigenetic age were largely explained by lifestyle-related confounders [11]. In a study of 3567 participants, the associations of moderate-to-vigorous activity with epigenetic age were U-shaped, and 1.5 h of moderate-to-vigorous activity per day was associated with the lowest *GrimAge* [12].

This study included follow-up over a decade, allowing sufficient time for changes in physical activity and epigenetic age to occur. Compared with previous research, it had a larger sample size, used more up-to-date and reliable measures of epigenetic age, and longitudinal associations were assessed. In addition, we considered the potential non-linearity of associations, for physical activity scores and confounding factors. The MCCS participants were overall healthier than the general Australian population [49], which probably involves higher levels of physical activity and a better epigenetic ageing profile. The longitudinal analyses involved a subset of participants who were randomly selected from controls in the nested cancer case–control studies, hence were reasonably representative of the overall cohort. They never had to attend follow-up to be selected, altogether contributing to a high attrition rate that might have affected the generalisability of our findings to less healthy populations. Another limitation is that physical activity was assessed using different questionnaires at baseline (frequency in times/week) and follow-up (duration in hours/week). While it was not possible to accurately compare physical activity at baseline and follow-up, the scores (i) had the same weighting for each type of activity included and (ii) were standardised to *z*-scores before subtraction, so their change can be interpreted as the change relative to the rest of the cohort. Physical activity data were obtained via self-reported questionnaires, whereas objective measures using accelerometers or pedometers could provide more valid and reliable measures. Previous studies using questionnaires [11–13] reported a weaker association between physical activity and epigenetic age than those using accelerometer-derived measures [14]. Measurement error usually biases association estimates towards the null, which may have contributed to explain the weak associations observed in our study. Previous studies in a similar setting reported an overestimation of physical activity when using questionnaires [50, 51]. Nevertheless, several studies reported that the IPAQ questionnaires used in our study provided fairly reliable and valid assessments of physical activity in older adults [52, 53]. Future studies could benefit from integrating quantitative bias analyses when using self-reports, or from obtaining more accurate measurement of physical activity, using device-based or combined assessments [54, 55].

In conclusion, we found a weak association of physical activity with epigenetic age. Additional studies with objective measures of physical activity and additional time points would be valuable to assess the benefits of physical activity on biological ageing.

## Supplementary Information

Below is the link to the electronic supplementary material.

Supplementary file1 (DOCX 795 KB)

## References

[1] Roychowdhury D. Using physical activity to enhance health outcomes across the life span. J Funct Morphol Kinesiol. 2020;5(1):2. 33467218 10.3390/jfmk5010002 PMC7739320

[2] Booth FW, Roberts CK, Laye MJ. Lack of exercise is a major cause of chronic diseases. Compr Physiol. 2012;2(2):1143–211. 23798298 10.1002/cphy.c110025 PMC4241367

[3] Moreno-Agostino D, Daskalopoulou C, Wu Y-T, et al. The impact of physical activity on healthy ageing trajectories: evidence from eight cohort studies. Int J Behav Nutr Phys Act. 2020;17(1):92. 32677960 10.1186/s12966-020-00995-8 PMC7364650

[4] López-Otín C, Blasco MA, Partridge L, et al. The hallmarks of aging. Cell. 2013;153(6):1194–17. https://linkinghub.elsevier.com/retrieve/pii/S0092867413006454. 10.1016/j.cell.2013.05.039 PMC3836174 23746838

[5] Elliott ML, Belsky DW, Knodt AR, et al. Brain-age in midlife is associated with accelerated biological aging and cognitive decline in a longitudinal birth cohort. Mol Psychiatry. 2021;26(8):3829–38. 31822815 10.1038/s41380-019-0626-7 PMC7282987

[6] Li A, Koch Z, Ideker T. Epigenetic aging: biological age prediction and informing a mechanistic theory of aging. J Intern Med. 2022;292(5):733–44. 35726002 10.1111/joim.13533

[7] Horvath S, Raj K. DNA methylation-based biomarkers and the epigenetic clock theory of ageing. Nat Rev Genet. 2018;19(6):371–84. http://www.nature.com/articles/s41576-018-0004-3. 10.1038/s41576-018-0004-3 29643443

[8] Dugué P-A, Bassett JK, Joo JE, et al. DNA methylation-based biological aging and cancer risk and survival: pooled analysis of seven prospective studies. Int J Cancer. 2018;142(8):1661–9. 10.1002/ijc.31189(1097-0215. 10.1002/ijc.31189 29197076

[9] Hillary RF, Stevenson AJ, McCartney DL, et al. Epigenetic measures of ageing predict the prevalence and incidence of leading causes of death and disease burden. Clin Epigenetics. 2020;12(1):115. 32736664 10.1186/s13148-020-00905-6 PMC7394682

[10] Dugué P-A, Bassett JK, Wong EM, et al. Biological aging measures based on blood DNA methylation and risk of cancer: a prospective study. JNCI Cancer Spectr. 2021;5(1):pkaa109. 10.1093/jncics/pkaa109/5983334. 33442664 10.1093/jncics/pkaa109 PMC7791618

[11] Kresovich JK, Garval EL, Lopez AMM, et al. Associations of body composition and physical activity level with multiple measures of epigenetic age acceleration. Am J Epidemiol. 2020;190(6):984–93. 10.1093/aje/kwaa251 PMC8168202 33693587

[12] Fox FAU, Liu D, Breteler MMB, et al. Physical activity is associated with slower epigenetic ageing—findings from the Rhineland study. Aging Cell. 2023;22(6):e13828. 37036021 10.1111/acel.13828 PMC10265180

[13] Kankaanpää A, Tolvanen A, Bollepalli S, et al. Leisure-time and occupational physical activity associates differently with epigenetic aging. Med Sci Sports Exerc. 2021;53(3):487–95. 32868581 10.1249/MSS.0000000000002498 PMC7886335

[14] Spartano NL, Wang R, Yang Q, et al. Association of accelerometer-measured physical activity and sedentary time with epigenetic markers of aging. Med Sci Sports Exerc. 2023;55(2):264–72. 36107108 10.1249/MSS.0000000000003041 PMC9840651

[15] Fiorito G, McCrory C, Robinson O, et al. Socioeconomic position, lifestyle habits and biomarkers of epigenetic aging: a multi-cohort analysis. Aging (Albany NY). 2019;11(7):2045–70. 31009935 10.18632/aging.101900 PMC6503871

[16] Dugué P-A, Bassett JK, Joo JE, et al. Association of DNA methylation-based biological age with health risk factors and overall and cause-specific mortality. Am J Epidemiol. 2017;187(3):529–38. 10.1093/aje/kwx291 29020168

[17] Robinson O, Chadeau Hyam M, Karaman I, et al. Determinants of accelerated metabolomic and epigenetic aging in a UK cohort. Aging Cell. 2020;19(6):e13149. 32363781 10.1111/acel.13149 PMC7294785

[18] Fiorito G, Caini S, Palli D, et al. DNA methylation‐based biomarkers of aging were slowed down in a two‐year diet and physical activity intervention trial: the DAMA study. Aging Cell 2021;20(10). 10.1111/acel.13439. 10.1111/acel.13439 PMC8520727 34535961

[19] Higgins-Chen AT, Thrush KL, Wang Y, et al. A computational solution for bolstering reliability of epigenetic clocks: implications for clinical trials and longitudinal tracking. Nature Aging. 2022;2(7):644–61. 36277076 10.1038/s43587-022-00248-2 PMC9586209

[20] Bernabeu E, McCartney DL, Gadd DA, et al. Refining epigenetic prediction of chronological and biological age. Genome Med. 2023;15(1):12. 36855161 10.1186/s13073-023-01161-y PMC9976489

[21] McGreevy KM, Radak Z, Torma F, et al. DNAmFitAge: biological age indicator incorporating physical fitness. Aging (Albany NY). 2023;15(10):3904–38. 36812475 10.18632/aging.204538 PMC10258016

[22] Belsky DW, Caspi A, Corcoran DL, et al. DunedinPACE, a DNA methylation biomarker of the pace of aging. eLife. 2022;11:e73420. https://elifesciences.org/articles/73420. 10.7554/eLife.73420 PMC8853656 35029144

[23] Azevedo MR, Araújo CLP, Reichert FF, et al. Gender differences in leisure-time physical activity. Int J Public Health. 2007;52(1):8. 17966815 10.1007/s00038-006-5062-1 PMC2778720

[24] Hägg S, Jylhävä J. Sex differences in biological aging with a focus on human studies. eLife. 2021;10:e63425. 33982659 10.7554/eLife.63425 PMC8118651

[25] Wrobel J, Muschelli J, Leroux A. Diurnal physical activity patterns across ages in a large UK based cohort: the UK biobank study. Sensors. 2021;21(4):1545. 33672201 10.3390/s21041545 PMC7927049

[26] Carrasquilla GD, García-Ureña M, F ll T, et al. Mendelian randomization a suggests a bidirectional, causal relationship between physical inactivity and adiposity. Elife 2022;11. 10.7554/eLife.70386. 10.7554/eLife.70386 PMC8975550 35254260

[27] Milne RL, Fletcher AS, MacInnis RJ, et al. Cohort profile: the Melbourne collaborative cohort study (Health 2020). Int J Epidemiol. 2017;46(6):1757–57i. http://academic.oup.com/ije/article/46/6/1757/3882696. 10.1093/ije/dyx085 28641380

[28] Sjostrom M, Ainsworth BE, Bauman A, et al. Guidelines for data processing analysis of the International Physical Activity Questionnaire (IPAQ) - Short and long forms. 2005.

[29] Ainsworth BE, Haskell WL, Leon AS, et al. Compendium of physical activities: classification of energy costs of human physical activities. Med Sci Sports Exerc. 1993;25(1):71–80. 8292105 10.1249/00005768-199301000-00011

[30] Jetté M, Sidney K, Blümchen G. Metabolic equivalents (METS) in exercise testing, exercise prescription, and evaluation of functional capacity. Clin Cardiol. 1990;13(8):555–65. 2204507 10.1002/clc.4960130809

[31] Dugué PA, Wilson R, Lehne B, Jayasekara H, Wang X, Jung CH, Joo JE, Makalic E, Schmidt DF, Baglietto L, Severi G. Alcohol consumption is associated with widespread changes in blood DNA methylation: analysis of cross‐sectional and longitudinal data. Addict biol. 2021;26(1):e12855. 10.1111/adb.12855 10.1111/adb.12855 31789449

[32] Geurts YM, Dugue PA, Joo JE, Makalic E, Jung CH, Guan W, Nguyen S, Grove ML, Wong EM, Hodge AM, Bassett JK. Novel associations between blood DNA methylation and body mass index in middle-aged and older adults. Int J Obes. 2018;42(4):887–96. 10.1038/ijo.2017.269 10.1038/ijo.2017.269 29278407

[33] Levine ME, Lu AT, Quach A, et al. An epigenetic biomarker of aging for lifespan and healthspan. Aging. 2018;10(4):573–91. 10.18632/aging.101414. 29676998 10.18632/aging.101414 PMC5940111

[34] Lu AT, Quach A, Wilson JG, et al. DNA methylation GrimAge strongly predicts lifespan and healthspan. Aging. 2019;11(2):303–27. 10.18632/aging.101684. 30669119 10.18632/aging.101684 PMC6366976

[35] Xu Z, Niu L, Kresvich JK, et al. Methscore: a comprehensive R function for DNA methylation-based health predictors. Bioinformatics. 2024. 10.1093/bioinformatics/btae302. 38702768 10.1093/bioinformatics/btae302 PMC11105949

[36] Australian Bureau of Statistics. Socio-economic indexes for areas (SEIFA), Australia. https://www.abs.gov.au/statistics/people/people-and-communities/socio-economic-indexes-areas-seifa-australia/latest-release. Accessed Oct 2024

[37] Stekhoven DJ, Buhlmann P. MissForest–non-parametric missing value imputation for mixed-type data. Bioinformatics. 2012;28(1):112–8. 10.1093/bioinformatics/btr597. 22039212 10.1093/bioinformatics/btr597

[38] Penone C, Davidson AD, Shoemaker KT, et al. Imputation of missing data in life-history trait datasets: which approach performs the best? Methods Ecol Evol. 2014;5(9):961–70.

[39] Li DL, Hodge AM, Cribb L, et al. Body size, diet quality, and epigenetic aging: cross-sectional and longitudinal analyses. J Gerontol: Ser A. 2024;79(4):glae026. 10.1093/gerona/glae026 PMC10953795 38267386

[40] Visontay R, Sunderland M, Slade T, et al. Are there non-linear relationships between alcohol consumption and long-term health?: a systematic review of observational studies employing approaches to improve causal inference. BMC Med Res Methodol. 2022;22(1):16. 35027007 10.1186/s12874-021-01486-5 PMC8759175

[41] Heydari G, Hosseini M, Yousefifard M, et al. Smoking and physical activity in healthy adults: a cross-sectional study in Tehran. Tanaffos. 2015;14(4):238–45. 27114725 PMC4841990

[42] Wadolowska L, Kowalkowska J, Lonnie M, et al. Associations between physical activity patterns and dietary patterns in a representative sample of Polish girls aged 13–21 years: a cross-sectional study (GEBaHealth Project). BMC Public Health. 2016;16(1):698. 27485607 10.1186/s12889-016-3367-4 PMC4971681

[43] Elliot CA, Hamlin MJ. Combined diet and physical activity is better than diet or physical activity alone at improving health outcomes for patients in New Zealand’s primary care intervention. BMC Public Health. 2018;18(1):230. 29422040 10.1186/s12889-018-5152-z PMC5806358

[44] Ball K, Crawford D, Owen N. Obesity as a barrier to physical activity. Aust N Z J Public Health. 2000;24(3):331–3. 10937415 10.1111/j.1467-842x.2000.tb01579.x

[45] Swain CTV, Drummond AE, Milne RL, et al. Linking physical activity to breast cancer risk via inflammation, part 1: the effect of physical activity on inflammation. Cancer Epidemiol Biomark Prev. 2023;32(5):588–96. 10.1158/1055-9965.EPI-22-0928 PMC10150243 36867865

[46] Lou MWC, Drummond AE, Swain CTV, et al. Linking physical activity to breast cancer via inflammation, part 2: the effect of inflammation on breast cancer risk. Cancer Epidemiol Biomark Prev. 2023;32(5):597–605. 10.1158/1055-9965.EPI-22-0929 PMC10150245 36867866

[47] Torma F, Kerepesi C, Jókai M, et al. Alterations of the gut microbiome are associated with epigenetic age acceleration and physical fitness. Aging Cell. 2024;23(4):e14101. 38414315 10.1111/acel.14101 PMC11019127

[48] Jokai M, Torma F, McGreevy KM, et al. DNA methylation clock DNAmFitAge shows regular exercise is associated with slower aging and systemic adaptation. GeroScience. 2023;45(5):2805–17. 37209203 10.1007/s11357-023-00826-1 PMC10643800

[49] Giles GG, English DR. The Melbourne Collaborative Cohort Study. IARC Sci Publ. 2002;156:69–70. 12484128

[50] Mahmood S, Nguyen NH, Bassett JK, et al. A quantitative bias analysis to estimate measurement error-related attenuation of the association between self-reported physical activity and colorectal cancer risk. Int J Epidemiol. 2020;49(1):153–61. 31687751 10.1093/ije/dyz209

[51] Bassett JK, Swain CTV, Hodge AM, et al. Calibration of the Active Australia questionnaire and application to a logistic regression model. J Sci Med Sport. 2021;24(5):474–80. 33281094 10.1016/j.jsams.2020.11.007

[52] Tomioka K, Iwamoto J, Saeki K, et al. Reliability and validity of the International Physical Activity Questionnaire (IPAQ) in elderly adults: the Fujiwara-kyo study. J Epidemiol. 2011;21(6):459–65. 21946625 10.2188/jea.JE20110003 PMC3899462

[53] Craig CL, Marshall AL, Sjostrom M, et al. International physical activity questionnaire: 12-country reliability and validity. Med Sci Sports Exerc. 2003;35(8):1381–95. 12900694 10.1249/01.MSS.0000078924.61453.FB

[54] Lynch BM, Dixon-Suen SC, Ramirez Varela A, et al. Approaches to improve causal inference in physical activity epidemiology. J Phys Act Health. 2020;17(1):80–4. 31810066 10.1123/jpah.2019-0515

[55] Sylvia LG, Bernstein EE, Hubbard JL, et al. Practical guide to measuring physical activity. J Acad Nutr Diet. 2014;114(2):199–208. 24290836 10.1016/j.jand.2013.09.018 PMC3915355

