# Supplementary material for: Physical activity and DNA methylation–based markers of ageing in 6208 middle-aged and older Australians: cross-sectional and longitudinal analyses
Source: GeroScience. 2024 Nov 7;47(2):2263–74. doi: 10.1007/s11357-024-01408-5 (PMC11979085; doi:10.1007/s11357-024-01408-5)
Supplement: Supplementary file 1 — Supplementary file1 (DOCX 795 KB) [file 11357_2024_1408_MOESM1_ESM.docx]

**Supplementary materials**

**Supplementary Figure 1.** Flow chart for the study sample selection in the cross-sectional and longitudinal analyses.


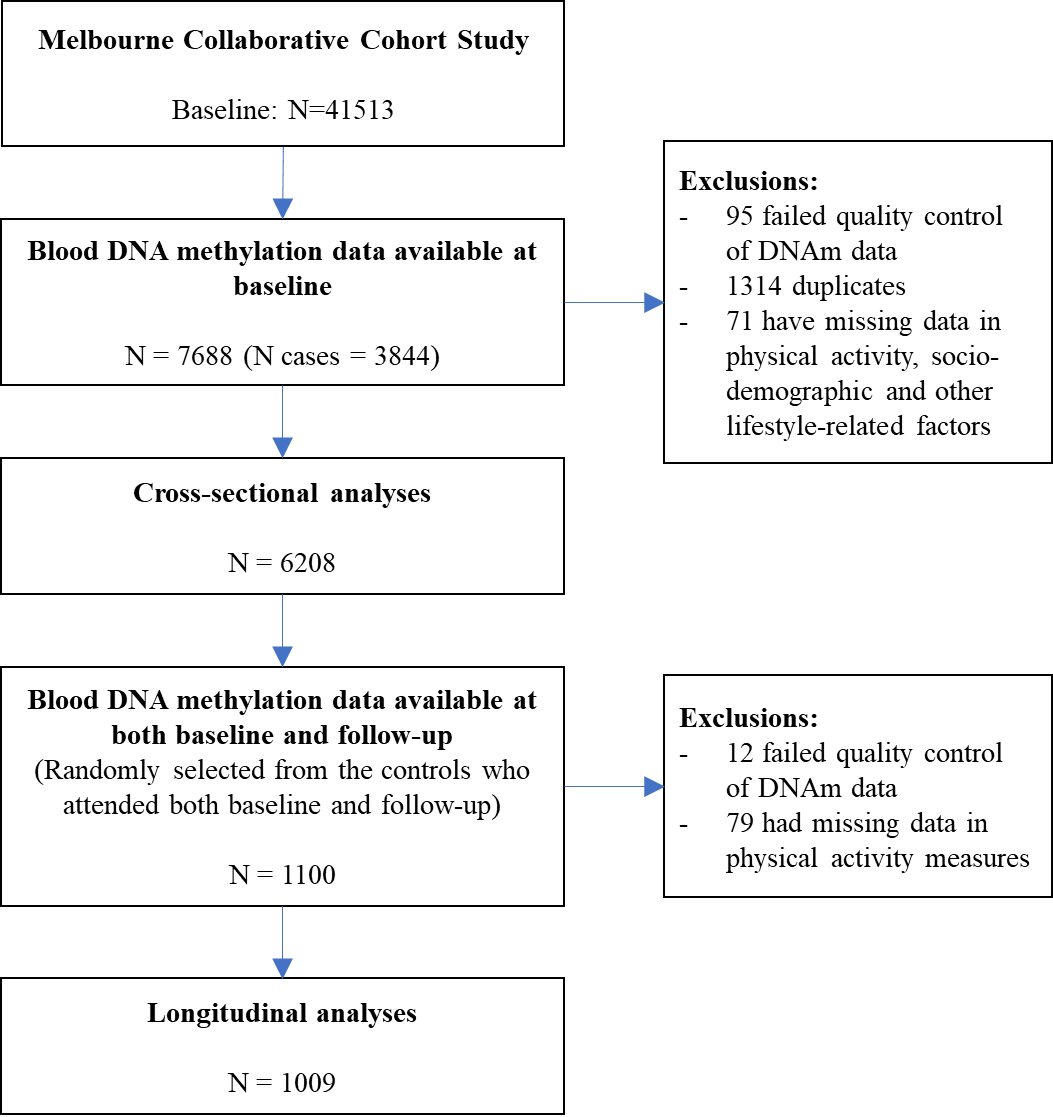


**Supplementary Figure 2.** Correlation matrix for baseline physical activity measures and epigenetic age (N=6208).


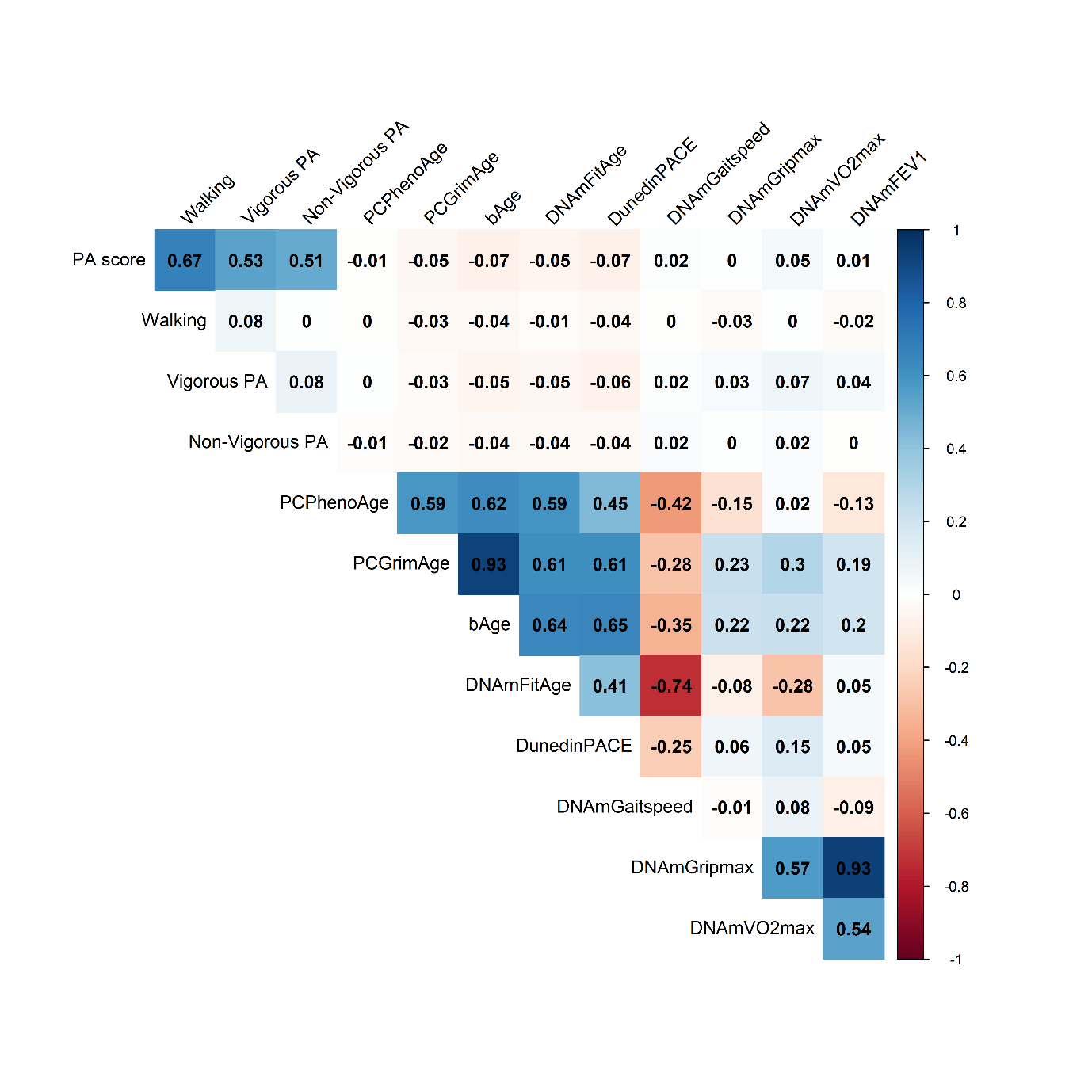


*All epigenetic ageing measures were age-adjusted.

**All physical activity variables were log-transformed.

**Supplementary Figure 3.**Correlation matrix for follow-up physical activity measures and epigenetic age (N=1008).


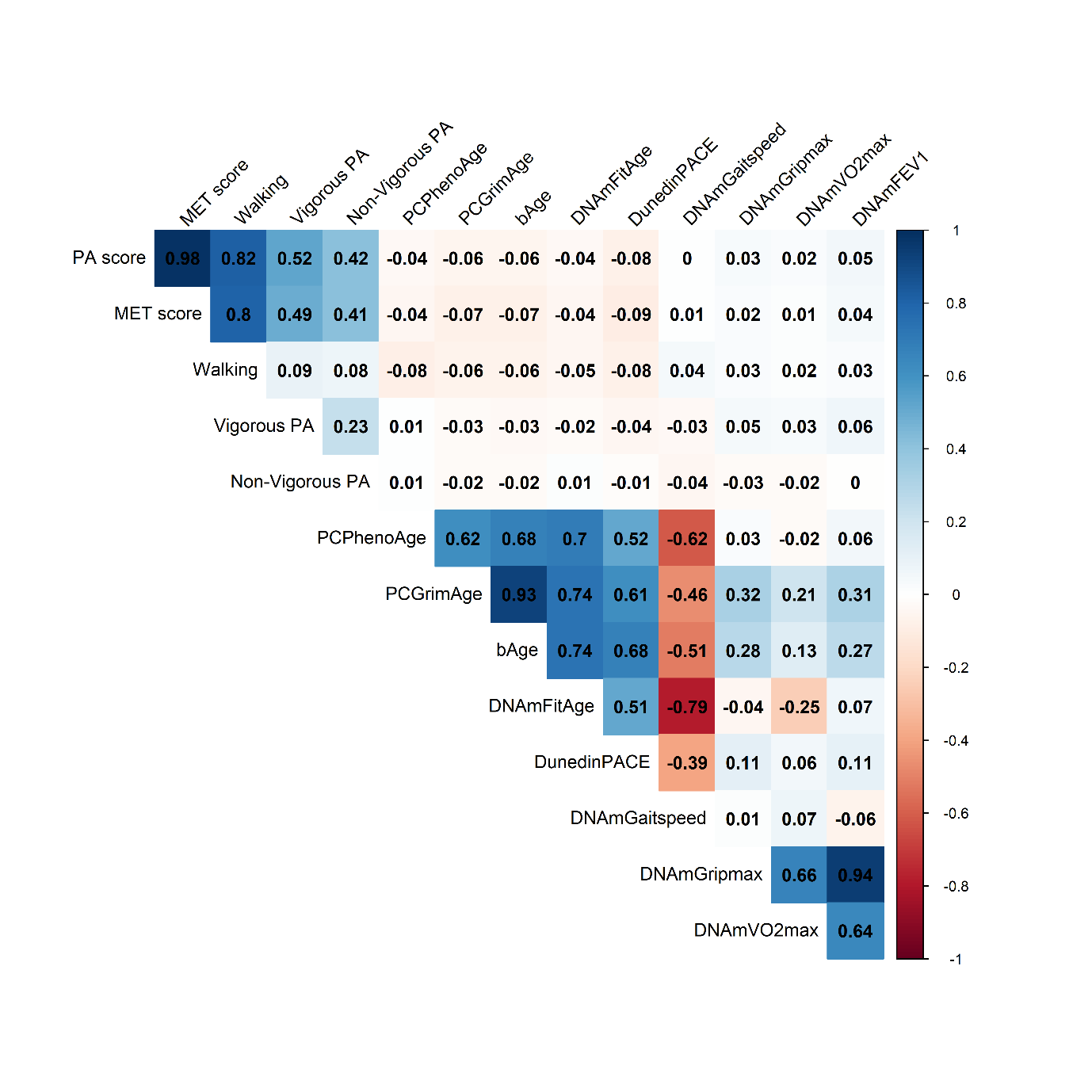


*All epigenetic ageing measures were age-adjusted.

**All physical activity variables were log-transformed.

**Supplementary Table 1.** Proportion of missing data at follow-up (N=1009).

| Variables | Missing | Percent |
| --- | --- | --- |
| AHEI-2010 | 71 | 7.0% |
| BMI | 1 | 0.1% |
| Alcohol consumption | 11 | 1.1% |
| SEIFA score | 38 | 3.8% |

**Supplementary Table 2.** Descriptive statistics for crude measures (not adjusted for age) of epigenetic age and epigenetic predictors of fitness markers.

| **Crude epigenetic predictors of ageing and fitness markers** | **Cross-sectional analysis (N=6208)** | **Longitudinal analysis (N=1009)** | |
| --- | --- | --- | --- |
|  | **Baseline** | **Baseline** | **Follow-up** |
| ***PCPhenoAge*, mean (SD)** | 50.92 (8.99) | 48.94 (8.69) | 59.82 (9.70) |
| ***PCGrimAge*, mean (SD)** | 67.32 (6.95) | 66.13 (7.10) | 75.74 (7.56) |
| ***bAge*, mean (SD)** | -0.10 (0.95) | -0.30 (0.98) | -0.02 (0.99) |
| ***DNAmFitAge*, mean (SD)** | 64.06 (8.69) | 61.61 (8.75) | 72.79 (9.06) |
| ***DunedinPACE*, mean (SD)** | 0.94 (0.13) | 0.92 (0.12) | 0.99 (0.12) |
| ***DNAmGaitspeed*, mean (SD)** | 1.84 (0.13) | 1.86 (0.13) | 1.75 (0.12) |
| ***DNAmGripmax*, mean (SD)** | 37.51 (7.95) | 39.61 (7.62) | 36.98 (7.65) |
| ***DNAmVO2max*, mean (SD)** | 39.44 (2.53) | 40.44 (2.19) | 38.91 (2.10) |
| ***DNAmFEV1*, mean (SD)** | 3.09 (0.61) | 3.27 (0.59) | 2.80 (0.50) |

*IQR = interquartile range; SD = standard deviation; SEIFA = socioeconomic index for areas; BMI = body mass index; AHEI-2010 = Alternative Healthy Eating Index 2010; MET = Metabolic Equivalent of Task; VO2amx = maximum volume of oxygen consumption; FEV1 = forced expiratory volume in 1 second.

**Supplementary Table 3.** Cross-sectional associations of different types of physical activity with epigenetic age.

| Waves | Types of activity | Epigenetic ageing | Model 1^a^ | | | Model 2^b^ | | | Model 3^c^ | | |
| --- | --- | --- | --- | --- | --- | --- | --- | --- | --- | --- | --- |
|  |  |  | β | 95% CI | P | β | 95% CI | P | β | 95% CI | P |
| Baseline  N=6208 | Walking | *PCPhenoAge* | -0.003 | -0.03, 0.02 | 0.84 | -0.01 | -0.03, 0.02 | 0.54 | -0.01 | -0.03, 0.02 | 0.68 |
|  |  | *PCGrimAge* | -0.03 | -0.05, -0.00 | **0.03** | -0.02 | -0.04, 0.01 | 0.14 | -0.01 | -0.04, 0.01 | 0.17 |
|  |  | *bAge* | -0.03 | -0.05, -0.01 | **0.02** | -0.02 | -0.04, 0.01 | 0.15 | -0.01 | -0.03, 0.01 | 0.23 |
|  |  | *DNAmFitAge* | 0.001 | -0.02, 0.03 | 0.94 | 0.01 | -0.01, 0.03 | 0.36 | 0.01 | -0.01, 0.04 | 0.30 |
|  |  | *DunedinPACE* | -0.03 | -0.05, -0.00 | **0.02** | -0.02 | -0.04, 0.00 | 0.12 | -0.01 | -0.03, 0.01 | 0.37 |
|  | Non-vigorous | *PCPhenoAge* | -0.01 | -0.04, 0.01 | 0.29 | -0.01 | -0.04, 0.01 | 0.28 | -0.01 | -0.04, 0.01 | 0.36 |
|  |  | *PCGrimAge* | -0.02 | -0.05, 0.00 | 0.08 | -0.004 | -0.02, 0.02 | 0.73 | -0.003 | -0.02, 0.02 | 0.79 |
|  |  | *bAge* | -0.03 | -0.05, -0.00 | **0.02** | -0.01 | -0.03, 0.01 | 0.46 | -0.01 | -0.03, 0.02 | 0.61 |
|  |  | *DNAmFitAge* | -0.02 | -0.04, 0.01 | 0.13 | -0.01 | -0.03, 0.02 | 0.54 | -0.01 | -0.03, 0.02 | 0.62 |
|  |  | *DunedinPACE* | -0.02 | -0.05, 0.00 | 0.06 | -0.01 | -0.03, 0.01 | 0.44 | -0.001 | -0.02, 0.02 | 0.90 |
|  | Vigorous | *PCPhenoAge* | -0.01 | -0.03, 0.02 | 0.63 | -0.005 | -0.03, 0.02 | 0.73 | -0.001 | -0.03, 0.03 | 0.97 |
|  |  | *PCGrimAge* | -0.05 | -0.08, -0.03 | **2×10^-5^** | -0.02 | -0.04, 0.00 | 0.12 | -0.02 | -0.04, 0.01 | 0.16 |
|  |  | *bAge* | -0.07 | -0.09, -0.04 | **9×10^-8^** | -0.02 | -0.05, -0.00 | **0.03** | -0.02 | -0.04, 0.00 | 0.06 |
|  |  | *DNAmFitAge* | -0.04 | -0.07, -0.02 | **0.002** | -0.01 | -0.03, 0.02 | 0.47 | -0.01 | -0.03, 0.02 | 0.57 |
|  |  | *DunedinPACE* | -0.06 | -0.09, -0.04 | **6×10^-7^** | -0.03 | -0.05, -0.01 | **0.01** | -0.02 | -0.04, 0.00 | 0.10 |
| Follow-up  N=1009 | Walking | *PCPhenoAge* | -0.08 | -0.14, -0.01 | **0.02** | -0.05 | -0.11, 0.01 | 0.08 | -0.05 | -0.11, 0.01 | 0.13 |
|  |  | *PCGrimAge* | -0.07 | -0.12, -0.01 | **0.02** | -0.02 | -0.07, 0.02 | 0.31 | -0.02 | -0.07, 0.03 | 0.41 |
|  |  | *bAge* | -0.06 | -0.12, -0.00 | **0.04** | -0.02 | -0.06, 0.03 | 0.51 | -0.01 | -0.06, 0.04 | 0.75 |
|  |  | *DNAmFitAge* | -0.04 | -0.10, 0.02 | 0.17 | -0.01 | -0.07, 0.04 | 0.65 | -0.01 | -0.06, 0.05 | 0.83 |
|  |  | *DunedinPACE* | -0.07 | -0.13, -0.01 | **0.03** | -0.03 | -0.09, 0.02 | 0.27 | -0.01 | -0.07, 0.05 | 0.71 |
|  | Non-vigorous | *PCPhenoAge* | 0.02 | -0.05, 0.08 | 0.61 | 0.01 | -0.05, 0.07 | 0.77 | 0.01 | -0.05, 0.07 | 0.70 |
|  |  | *PCGrimAge* | -0.01 | -0.06, 0.05 | 0.77 | -0.02 | -0.07, 0.03 | 0.36 | -0.02 | -0.07, 0.03 | 0.39 |
|  |  | *bAge* | -0.01 | -0.07, 0.05 | 0.78 | -0.02 | -0.07, 0.03 | 0.38 | -0.02 | -0.07, 0.03 | 0.44 |
|  |  | *DNAmFitAge* | 0.02 | -0.04, 0.08 | 0.47 | 0.01 | -0.05, 0.07 | 0.71 | 0.01 | -0.04, 0.07 | 0.65 |
|  |  | *DunedinPACE* | 0.003 | -0.06, 0.06 | 0.92 | -0.001 | -0.06, 0.06 | 0.98 | 0.01 | -0.05, 0.06 | 0.80 |
|  | Vigorous | *PCPhenoAge* | 0.01 | -0.05, 0.07 | 0.75 | 0.02 | -0.04, 0.08 | 0.59 | 0.02 | -0.04, 0.08 | 0.49 |
|  |  | *PCGrimAge* | -0.04 | -0.10, 0.01 | 0.14 | -0.01 | -0.06, 0.04 | 0.69 | -0.01 | -0.06, 0.04 | 0.76 |
|  |  | *bAge* | -0.04 | -0.10, 0.02 | 0.15 | -0.01 | -0.06, 0.04 | 0.79 | -0.002 | -0.05, 0.05 | 0.94 |
|  |  | *DNAmFitAge* | -0.02 | -0.08, 0.04 | 0.53 | 0.001 | -0.06, 0.06 | 0.97 | 0.005 | -0.05, 0.06 | 0.87 |
|  |  | *DunedinPACE* | -0.04 | -0.10, 0.02 | 0.20 | -0.01 | -0.07, 0.04 | 0.64 | -0.002 | -0.06, 0.05 | 0.95 |

^a^Model 1 adjusted for age, sex, and country of birth; ^b^Model 2 additionally adjusted for SEIFA score, smoking status, and pack-years, AHEI-2010, and alcohol consumption (spline term); ^c^Model 3 additionally adjusted for BMI (spline term).

*All epigenetic ageing measures were age-adjusted, and were standardised to a mean of 0 and standard deviation of 1.

**At baseline, each type of physical activity was recorded as frequency, i.e. times per week, and was scored as 0 = No activity, 1.5 = 1-2 times per week; 4 = ≥ 3 times per week; at follow-up, they were recorded as time spend on each activity, i.e. hours per week. At both time points, physical activity variables were log-transformed and standardised to a mean of 0 and standard deviation of 1
